# Integrating molecular methods and biophysical modeling to assess functional connectivity between marine protected areas

**DOI:** 10.1002/eap.70150

**Published:** 2025-12-08

**Authors:** Kingsly C. Beng, Anna Akimova, Silke Laakmann, Vera Sidorenko, Sara Rubinetti, Santiago E. A. Pineda‐Metz, Bernadette Pogoda, Sarah C. Brand, Kerstin Klemm, K. Mathias Wegner, Lisa N. S. Shama, Lara Schmittmann, Luis Gimenez, Katharina Alter, Brecht Stechele, Amin Rahdarian, Christian Winter, Alexey Androsov, Inna Sokolova, Anne F. Sell

**Affiliations:** ^1^ Thünen Institute of Sea Fisheries Bremerhaven Germany; ^2^ Helmholtz Institute for Functional Marine Biodiversity at the University of Oldenburg (HIFMB) Oldenburg Germany; ^3^ Alfred Wegener Institute, Helmholtz Centre for Polar and Marine Research (AWI) Bremerhaven Germany; ^4^ Alfred Wegener Institute, Helmholtz Centre for Polar and Marine Research (AWI) Wadden Sea Station Sylt Germany; ^5^ Department for Sustainable Development and Ecological Transition University of Piemonte Orientale Vercelli Italy; ^6^ GEOMAR Helmholtz Centre for Ocean Research Kiel Kiel Germany; ^7^ School of Ocean Sciences, Bangor University Bangor UK; ^8^ Alfred Wegener Institute, Helmholtz Centre for Polar and Marine Research (AWI), Biologische Anstalt Helgoland Germany; ^9^ Department of Coastal Systems Royal Netherlands Institute for Sea Research (NIOZ) Den Burg The Netherlands; ^10^ Department of Marine Biology Institute for Biological Sciences, University of Rostock Rostock Germany; ^11^ Royal Belgian Institute of Natural Sciences Brussel Belgium; ^12^ Institute of Geosciences, Kiel University Kiel Germany; ^13^ Department of Maritime Systems, Interdisciplinary Faculty University of Rostock Rostock Germany

**Keywords:** ecosystem restoration, environmental DNA, Lagrangian simulation, Natura 2000 sites, oyster reefs, zooplankton

## Abstract

Marine protected area (MPA) networks are important for supporting biodiversity, enhancing ecosystem resilience, and facilitating species recovery. For the effectiveness of conservation and restoration, functional connectivity plays a vital role. The dispersal, movement, and successful establishment of organisms between suitable habitats and MPAs ensure long‐term sustainability of the populations. Despite its importance, functional connectivity is rarely integrated into restoration planning, which limits the effectiveness of species reintroductions, habitat connectivity, and adaptation to environmental changes. In this study, we applied an integrative approach combining molecular detections (environmental DNA [eDNA] and meroplankton metabarcoding) with biophysical modeling to explore the functional connectivity between two Natura 2000 MPAs in the North Sea: Borkum Reef Ground (BRG) and Sylt Outer Reef (SOR). We focused on the European flat oyster (*Ostrea edulis*), a reef‐building species that once provided vast reef habitats but is now functionally extinct in the German Bight and is therefore the subject of recent restoration measures at BRG. Our results showed partial but informative correspondence between molecular detections of oyster genetic traces and the modeled larval pathways during the June–July 2022 sampling period. We further explored larval dispersal across entire spawning seasons in 2022 and 2023. Connectivity between BRG and SOR was highly dependent on larval drift depth. Surface‐drifting larvae showed strong interannual variability, with 3% reaching SOR in 2022 when northwesterly winds dominated, increasing to 22% in 2023 under westerly and southwesterly winds. Larvae drifting at depth, however, exhibited near‐zero connectivity, leading to high self‐recruitment rates, with over 25% settling near the original restoration sites. Our results demonstrate that wind‐driven currents are a key driver of interannual variability in larval retention and dispersal. Additionally, they highlight the role of biological traits, such as vertical positioning and pelagic larval duration, in shaping connectivity between MPAs and oyster restoration sites. These findings emphasize the need to integrate connectivity assessments into MPA management and the restoration planning of reef‐building benthic species. The interdisciplinary approach presented here provides a quantitative framework for assessing connectivity under species‐ and site‐specific conditions, offering a transferable tool to evaluate the restoration potential of other species and enhance the functional network between MPAs.

## INTRODUCTION

Marine protected areas (MPAs) play a key role by offering refugia for biodiversity, safeguarding critical habitats, and maintaining ecological processes (Friesen et al., 2019; White et al., 2024). However, with intense past and current anthropogenic activities such as overexploitation and pollution, as well as climate change, isolated and individual MPAs may not be able to sufficiently counteract these complex and dynamic challenges. Therefore, there is an increasing emphasis to establish MPA networks that collectively enhance ecological resilience and functionality (Arafeh‐Dalmau et al., 2021; Hernández‐Andreu et al., 2024; Jonsson et al., 2020).

Connectivity, that is, the functional linkage between geographically distant habitats, determines the effectiveness of MPA networks (Cannizzo et al., 2021; Carr et al., 2017; Cook et al., 2014; Munroe et al., 2014). The movement of species across MPAs facilitates genetic exchange, which is critical for maintaining viable and resilient populations and contributes to the adaptability and long‐term survival of species in the face of environmental changes (Mackenzie et al., 2022; Wilcox et al., 2023). A spillover effect from a source site actively facilitates connectivity, wherein the benefits accrued within one MPA extend beyond its boundaries, enhancing ecosystem productivity and ecological health in adjacent areas (Lenihan et al., 2021, 2022). In addition, theoretical models show that connectivity promotes recovery from mass mortalities (such as have been recently reported in bivalves worldwide; Burdon et al., 2014; Soon & Ransangan, 2019) by enabling the recruitment of new organisms into other areas (Giménez et al., 2020). In sessile benthic species with a pelagic larval phase, larval behavior plays a significant role in dispersal and connectivity (Cowen & Sponaugle, 2009; Robins et al., 2013).

A well‐connected MPA network can provide refugia for endangered species such as the European flat oyster (*Ostrea edulis*). *O. edulis* was once abundant in European waters, including the Wadden Sea, German Bight, and English Channel, forming extensive reefs that provided valuable habitat and ecosystem services (Pineda‐Metz et al., 2023; Pogoda, 2019; Thurstan et al., 2024). As suspension feeders, oysters contribute to bentho‐pelagic coupling, improving water quality, and regulating nutrient dynamics (Dame et al., 2001; zu Ermgassen et al., 2024). Oyster reefs provide complex three‐dimensional structures that support diverse communities of marine organisms (Kregting et al., 2020; Zempléni et al., 2025). *O. edulis* abundance has declined during the past century due to overharvesting, habitat degradation, and disease (Lown et al., 2020; Pineda‐Metz et al., 2023; Pogoda et al., 2019, 2023; Stechele, Barbut, et al., 2023). The loss of oyster reefs has resulted in decreased water quality, reduced nutrient uptake, and diminished bentho‐pelagic coupling (Kemp et al., 2005; Pogoda, 2019; Ray & Fulweiler, 2020). In the Firth of Forth, Scotland, the loss of European flat oyster beds resulted in lower species richness throughout the 19th and early 20th centuries (Thurstan et al., 2013, 2024). Given the extensive historical distribution of *O. edulis* and its ecological importance in the North Sea, their disappearance most likely produced relevant (but yet unquantified) alterations to the food web.

Efforts are now underway to restore *O. edulis* populations in the Dutch, Belgian, and German North Sea, with the aim of enhancing biodiversity and ecosystem services (Pogoda, 2019; Pogoda et al., 2020). Borkum Reef Ground (BRG), Sylt Outer Reef (SOR), and Doggerbank are three German MPAs within the European Union's MPA network (designated as Natura 2000 sites) and Special Areas of Conservation (SAC) under the EU Habitats Directive due to the presence of the habitat type “reef” (EU code 1170) (European Commission et al., 2012). The sea floor conditions of BRG and SOR are characterized by glacial hard‐substrates as well as typical sandy bottoms (Hahn et al., 2022; Michaelis et al., 2020). The hard‐bottom environments can support a diverse reef community of sessile and mobile invertebrates, alongside various mobile vertebrate species, creating a distinct habitat within the North Sea (Hahn et al., 2022; Michaelis et al., 2020). A unique initiative in BRG compared to the other Natura 2000 sites is the presence of an *O. edulis* restoration program consisting of two pilot oyster reefs, established in 2020 (Pineda‐Metz et al., 2023). Successful oyster reef habitat restoration in BRG may facilitate the spreading of *O. edulis* beyond this MPA and eventually into other regions, including Natura 2000 sites in the North Sea. However, it has remained uncertain whether this actually happens, as factors such as larval dispersal distance, environmental conditions, and connectivity dynamics between habitats are not fully understood. In the Netherlands, two oyster restoration sites have been established in the direct vicinity of BRG, close to the border of the German exclusive economic zone, EEZ: (1) Gemini Buitengaats oyster bed, established in 2018–2019 and (2) Borkumse stenen oyster bed, established in 2018 (Bos et al., 2023). Previous studies have suggested that dispersal and settlement of *O. edulis* can be influenced by larval behavior, the timing of spawning, as well as by the size of the brood stock (Bertolini & Pastres, 2023; Guy et al., 2019; Rodriguez‐Perez et al., 2019). We investigated dispersal patterns and behavior of *O. edulis* larvae in the North Sea to provide important insights into the effectiveness of current restoration, conservation and management strategies in promoting connectivity of settlement sites and sustaining viable populations.

To investigate functional connectivity, evidence of *O. edulis* occurring inside and across the respective MPAs is needed. For the planktonic larvae the lack of diagnostic characters hampers the identification to species level based on morphology (Bracken‐Grissom et al., 2012; Laakmann et al., 2020; Pardo et al., 2009; Sweeney et al., 1992). Therefore, molecular genetic methods such as DNA metabarcoding, an approach that harnesses the power of DNA sequencing to identify multiple species simultaneously from bulk biological and environmental samples, have emerged as a revolutionary tool for efficiently and comprehensively identifying species and assessing biodiversity (Huang et al., 2022; Taberlet et al., 2012).

Complementary to observational methods, hydrodynamic modeling and biophysical modeling have emerged as powerful tools for studying and predicting connectivity between marine habitats (Abecasis et al., 2023; Andrello et al., 2013; Claro et al., 2019; Le Port et al., 2014; Sidorenko et al., 2025). Hydrodynamic models simulate ocean currents, temperature, salinity, and other physicochemical parameters in space and time (Fajardo‐Urbina et al., 2023; Sidorenko et al., 2025; Wang et al., 2022). These hydrodynamic simulations provide the physical parameters that biophysical models integrate to predict the dispersal of propagules (Akimova et al., 2016; Assis et al., 2021; Pastor Rollan et al., 2023). Such biophysical models have been successfully used to investigate factors influencing connectivity, such as larval stage duration, larval behavior, environmental conditions, and potential dispersal barriers (Benestan et al., 2021; Berglund et al., 2012).

The goal of this study was to combine molecular methods—DNA metabarcoding and quantitative PCR (qPCR)—with biophysical modeling to assess the functional connectivity between MPAs and restoration sites for the European flat oyster (*O. edulis*). Specifically, we aimed to determine the potential for spillover effects from currently restored native oyster reefs in BRG into surrounding habitats and to SOR through the dispersal of planktonic life stages. The DNA metabarcoding and qPCR techniques involved analyzing environmental DNA (eDNA) from sea water and from meroplankton bulk samples to detect the presence and distribution of *O. edulis* (in the case of bulk samples, the larvae) in the study area. This allowed us to confirm oyster occurrences, even at low densities. Biophysical modeling predicted dispersal patterns of planktonic oyster larvae, offering insights into potential connectivity between three restoration sites and a distant MPA, beyond the sampling period. We integrate these methodologies to gain a comprehensive understanding of the ecological status of *O. edulis*, validating our observations with model predictions and informing effective restoration efforts and conservation strategies.

## MATERIALS AND METHODS

### Study area and sample collection

**FIGURE 1:**
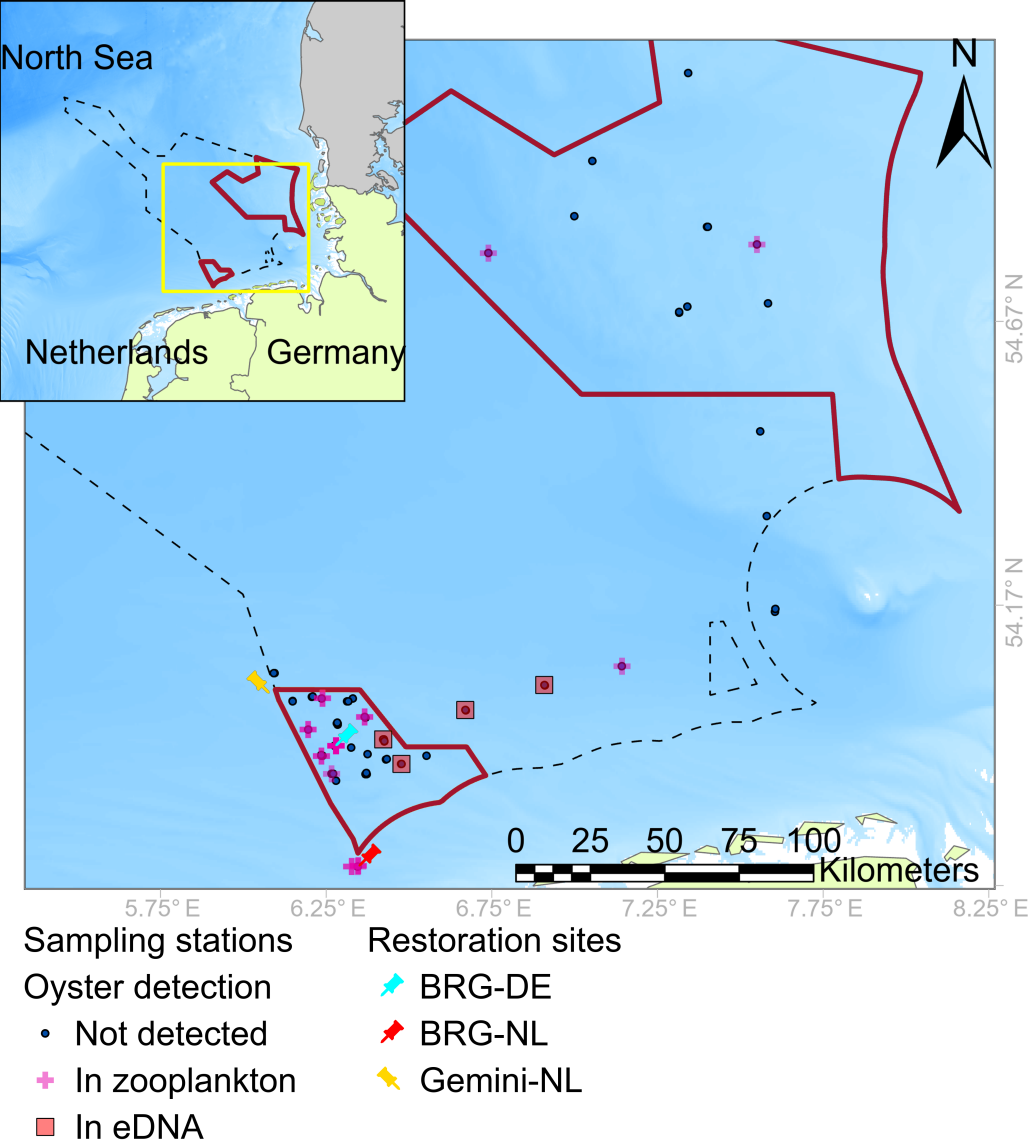
Locations of European flat oyster (*Ostrea edulis*) DNA detections (metabarcoding and quantitative PCR) in zooplankton and environmental DNA (eDNA) samples during the 2022 sampling campaign. The map highlights the two marine protected areas (MPAs): Borkum Reef Ground (BRG, southern) and Sylt Outer Reef (SOR, northern). Sampling stations are shown as blue dots when oysters were not detected, with magenta crosses indicating the presence of oyster DNA in meroplankton, and with orange squares representing eDNA detections. Restoration sites within the BRG include BRG‐DE (cyan marker), BRG‐NL (red marker), and Gemini‐NL (yellow marker). The inset map (top left) provides the regional context within the southeastern North Sea.

The study was conducted in the German Bight in the southern North Sea, with a focus on the Natura 2000 MPAs Borkum Reef Ground (BRG) and Sylt Outer Reef (SOR) in the German Exclusive Economic Zone (EEZ) (Figure 1). BRG covers an area of 625 km^2^, with water depths of 18–33 m, whereas SOR comprises 5321 km^2^, with water depths of 8–48 m (Hahn et al., 2022). The area between BRG and SOR (hereafter referred to as the transit region) was also included in the analysis of potential larval transport pathways. Three established *O. edulis* restoration sites are located in this region: the German site within BRG (BRG‐DE; 53.9166° N, 6.2802° E) and two Dutch sites, BRG‐NL (53.7017° N, 6.3490° E) and Gemini‐NL (54.0107° N, 6.0777° E) (Figure 1).

Meroplankton and eDNA sampling was conducted in the MPAs and transit regions in June–July 2022 during three cruises of the RV *Heincke* (HE601: AWI, 2017; Pineda‐Metz, 2022), FRV *Walther Herwig III* (WH457), and MS *Krebs Helios*, respectively. Fifty zooplankton samples were collected using a Baby Bongo net (frame 135 cm wide, 2 × 20 cm diameter, 250 cm long, 100 μm mesh size) in double‐oblique hauls at a ship speed of two knots. A 22 kg V‐Fin depressor (Scherfuss) was attached to the net, and the combination was deployed vertically into the water column at a wire speed of 0.7 m/s to a maximum depth of 10 m above the sea floor. The net was towed at two knots and simultaneously retrieved at 0.3–0.5 m/s. The contents of the net were then filtered through a sieve cascade (200 and 63 μm) and the resulting organisms were fixed in absolute ethanol. To avoid degradation, the samples were refixed after 24 h (by pouring the sample over a 63‐μm sieve and replacing the old ethanol with new ethanol) and stored at 4°C until further processing. On board MS *Krebs Helios*, zooplankton samples (*n* = 12) were collected using a pump coupled with a hose for targeted sampling in the direct vicinity of the restoration site (and at further stations). A weight was attached to the hose and the combination was deployed vertically into the water column to a depth of 1 m above the sea floor. The pump was switched on until 200 L of seawater was filtered through a sieve cascade (200, 63, and 53 μm), and the resulting filtrates were processed in the same way as the net samples.

For eDNA, seawater samples were collected at two depths using a CTD rosette equipped with 12 Niskin bottles. The Niskin bottles were deployed open and the first six were closed at the bottom (5 m above the seafloor) and the remaining six at the surface (5 m below the surface). Once the CTD rosette was on deck, 10 L each of bottom and surface seawater was collected into clean plastic canisters and filtered in the wet lab on board. Contamination prevention was conducted according to Ohnesorge et al. (2023). Similarly, filtration was performed according to Ohnesorge et al. (2023) in triplicates using a Rocker 400 oil‐free vacuum pump (Rocker Scientific Co., Ltd.) at 400 Pa. For each sample, 2 L of seawater was filtered through 0.45 μm pore size (Whatman, ø 47 mm) sterile nitrocellulose membrane filters. Subsequently, the filters were stored in sterile 2‐mL Eppendorf tubes containing 1.5 mL absolute ethanol and frozen at −20°C until further processing. At the end of each day, blanks consisting of distilled water were filtered and processed in the same way as the biological samples to check for potential contamination. Apart from the *Krebs Helios* cruise, during which eDNA samples were not collected, all eDNA and meroplankton samples were collected in parallel at the same stations. Overall, meroplankton and eDNA sampling was conducted at 40 stations across a depth range spanning 10–43 m.

### DNA extraction, library preparation, and sequencing

Prior to DNA extraction, meroplankton samples were split into two equal portions following Ohnesorge et al. (2023): one half for metabarcoding and the other half for potential morphological identification. Each sample was poured into a Motoda‐splitter (Motoda, 1959), and the splitter was rotated 20 times back and forth to separate the sample into two equal halves. The splitter was sterilized between samples by cleaning with 12% bleach, rinsing with distilled water and incubating under UV light for 15 min. DNA was extracted from eDNA and split meroplankton samples using DNeasy Blood and Tissue kits (Qiagen, Hilden Germany) and following a modified protocol (Ohnesorge et al., 2023). DNA was extracted in duplicate for zooplankton (*n* = 92) and from each of the triplicate filters for eDNA (*n* = 170), resulting in 262 samples.

Library preparation and sequencing were conducted according to Ohnesorge et al. (2023). All samples were amplified using universal primer sets mlCOIintF‐XT (Wangensteen et al., 2018) and jgHCO2198 (Geller et al., 2013) that target a 313 base pairs (bp) region of the mitochondrial cytochrome c oxidase subunit 1 (COI) gene of invertebrates. Libraries were sequenced on an Illumina MiSeq using a v3 sequencing kit (600 cycles). Each sequencing run consisted of 90 biological samples and six negative controls (from two sampling, extraction, and PCR blanks each). Raw sequence data (fastq.gz files) and sample metadata were deposited in the European Nucleotide Archive (ENA) at EMBL‐EBI under accession no. PRJEB98425 (https://www.ebi.ac.uk/ena/browser/view/PRJEB98425) using the data brokerage service of the German Federation for Biological Data GFBio (Diepenbroek et al., 2014) in compliance with the Minimal Information about any (X) Sequence (MIxS) standard (Yilmaz et al., 2011).

### Bioinformatics analysis

Raw sequence data were analyzed using DADA2 1.30.0 (Callahan et al., 2016) in R 4.3.2 (R Core Team, 2023). Cutadapt 4.1 (Martin, 2011) was used to remove primers and adapters, and the following parameters were used: truncate forward reads at position 250 and reverse reads at position 180, apply maximum expected error (EE) of 2.5 and 1.8 for forward and reverse reads, respectively. The remaining parameters were set to default. Taxonomic assignment of amplicon sequence variants (ASVs) was performed using two methods as follows: (1) with the COI MetaZooGene database (Bucklin et al., 2021) via the RDP Naive Bayesian Classifier (Wang et al., 2007) with bootstrap confidence minBoot = 80 and (2) with the NCBI GenBank database via Blast (Camacho et al., 2009) with default settings. Potential contamination was checked and removed by comparing the prevalence of each ASV in negative controls with biological samples using decontam (Davis et al., 2018).

### Species‐specific detection of *O. edulis*

Aside from metabarcoding, we applied qPCR as a targeted complementary technique to detect the presence of *O. edulis* in 215 DNA aliquots from 153 eDNA and 62 meroplankton samples. Specific primers and TaqMan probes targeting the 16S rRNA gene of *O. edulis* were used (Appendix S1: Table S1) along with general Bivalvia 18S rRNA primers (Sánchez et al., 2014). qPCR reactions were prepared in a total volume of 20 μL containing 10 μL of TaqMan Fast Universal PCR Master Mix (2×; Life Technologies), 0.24 or 0.72 μL of each primer, 0.16 or 0.18 μL of probe, 2 μL of DNA (5–10 ng/μL), and RNase‐free water (Life Technologies) as described in Sánchez et al. (2014). Each reaction was run in duplicate on a 7500 Fast Real‐Time PCR System (Applied Biosystems) with the following cycling profile: 95°C for 20 s, followed by 40 cycles of 95°C for 30 s and 60°C for 30 s. Three no‐template control (NTC) wells were included in all qPCR assays to rule out contamination, alongside positive control samples containing only *O. edulis* DNA. PCR efficiency was determined using five 10‐fold serial dilutions of *O. edulis* 16S and Bivalvia 18S DNA, starting at 100 ng.

### Water temperature and wind data

To provide an environmental context for our larval transport simulations in 2022 and 2023, we compared the long‐term mean values of temperature regime (1982–2021) and wind conditions (1975–2021) to the specific summer conditions observed in these two years. Wind data were retrieved from the ERA5 reanalysis dataset (10 m eastwards and northwards components; Hersbach et al., 2020, 2023) for the BRG area (Rubinetti et al., 2023). Daily water temperature was obtained from the North‐West European Shelf reanalysis from Copernicus Marine Service (https://doi.org/10.48670/moi-00059) and averaged over the German Bight (53–56° N and 3–9° E).

### Hydrodynamic and biophysical modeling

We used a Lagrangian particle module offline coupled to the Finite‐volume Sea‐ice Ocean Model Coastal branch (FESOM‐C) hydrodynamic model. The detailed description of the FESOM‐C setup is presented in Sidorenko et al. (2025). Here, we present only the key details and specific information about the model simulations relevant to the current study.

FESOM‐C is a coastal branch of the global sea ice ocean model FESOM (Androsov et al., 2019; Fofonova et al., 2021). It solves three‐dimensional (3D) primitive equations under Boussinesq, hydrostatic, and traditional approximations for momentum, continuity, and density, using a mixed unstructured mesh. The horizontal resolution of the model's grid varies between 30 and 100 m in the intertidal zone and approximately 1 km near the northern boundary of BRG. The applied 2D‐model setup simulates vertically averaged horizontal velocities, accounting for wind, atmospheric pressure, tidal forcing, and depth‐averaged baroclinic pressure gradients. Surface velocities were reconstructed from the depth‐averaged velocities employing a neural network approach trained by observed surface drifter pathways (Sidorenko et al., 2025). The particle drift integration step of the Lagrangian module was 465 s.

The total duration of larval drift was constrained by the known temperature‐dependent larval duration, following De Mesel et al. (2018) and Stechele, Hughes, et al. (2023):

(1) $${\mathrm{PLD}}_{\left(\text{days}\right)}=1025.315\times T^{-1.56}$$

where PLD is pelagic larval duration and *T* (in degrees Celsius) is the daily mean water temperature in the German Bight. For each of the simulations described below, we tested two larval drift duration scenarios. In the first, termed “strict temperature‐dependent pelagic larval duration” (strict PLD), the modeled larvae were assumed to settle immediately upon completing their pelagic stage as determined by Equation (1). In the second scenario, referred to as “extended PLD,” we considered the larvae's ability to delay metamorphosis while awaiting suitable settlement conditions. Rodriguez‐Perez et al. (2021) found that *O. edulis* larvae are capable of prolonging their pelagic stage by up to 14 days if conditions are unfavorable for their settlement. Therefore, we considered *O. edulis* larvae to be competent to settle on any day during this 2‐week period after the end of their initial pelagic stage given by Equation (1). These two approaches allowed us to explore how larval behavior could influence connectivity between *O. edulis* populations.

Information about vertical distributions of *O. edulis* larvae in the water column is very sparse and hence it is unknown whether larvae in the field travel near the surface, or in the deeper water layers. Wilson (1987) reported that larger larvae (≥250 μm) are evenly distributed throughout the water column, while Preston et al. (2020) found higher concentrations of smaller larvae (up to 190 μm) near the surface. To address uncertainties in larval vertical distribution during their drift, we tested two scenarios: (1) surface drift, using surface currents and temperatures to simulate larvae drifting at the surface and (2) depth‐averaged drift, using depth‐averaged currents and temperatures to represent larvae evenly distributed throughout the water column.

Using the biophysical model for these scenarios with regard to PLD and the vertical position, we conducted two sets of modeling experiments (Table 1). The first set represents the June–July 2022 sampling campaign (“sampling season”), specifically focusing on larval dispersal during June and July, and was intended for comparison with molecular detections during the research cruises in that period. The second set aimed to capture broader seasonal and interannual variability by simulating larval transport over the full spawning seasons of 2022 and 2023 termed “spawning season.” Within the spawning season simulations, we distinguished alternative locations for larval release: (1) the three current restoration sites in Germany and the Netherlands and (2) the entire area of the Natura 2000 site BRG, considering the possibility that oysters have or would spread within this MPA (Table 1).

**TABLE 1:** Overview of all model simulations and parameters used.

Larvae source	Sampling season	Spawning season	
	Three restoration sites	Three restoration sites	Entire BRG
Period	June 15–July 6	June 15–August 15	June 15–August 15
Year	2022	2022 and 2023	2022 and 2023
Surface drift	Yes	Yes	Yes
Depth‐averaged drift	Yes	Yes	Yes
Strict PLD PLD_(days)_ = 1025.315 × *T* ^−1.56^	Yes	Yes	Yes
Extended PLD PLD_(days)_ = 1025.315 × *T* ^−1.56^ + 14	Yes	Yes	Yes
Particles released per hour	200	200	500
No. seeding days	21	61	61
Modeling purpose	Comparison with molecular detections	Assessing connectivity to SOR and self‐recruitment	Assessing connectivity to SOR and self‐recruitment

Abbreviations: BRG, Borkum Reef Ground; PLD, pelagic larval duration; SOR, Sylt Outer Reef; T, temperature (in degrees Celsius).

#### Larval dispersal during the sampling season in 2022

Here, we investigated larval dispersal from the three *O. edulis* restoration sites. Model particles were released within a 1‐km radius around each restoration site to represent larvae originating from these areas during the sampling cruises in summer 2022 (Table 1). All released particles were allowed to drift until they either reached their maximal PLD or until the end of the cruises on July 6, resulting in a maximum drift duration of 3 weeks.

#### Larval dispersal during the spawning seasons in 2022 and 2023

To further investigate larval transport and connectivity among the three restoration sites within BRG and SOR, we expanded simulations to cover the entire *O. edulis* spawning seasons in 2022 and 2023. Spawning of *O. edulis* is typically initiated when water temperatures exceed 15°C (Bromley et al., 2016; Maathuis et al., 2020) and a cumulative thermal threshold of ~570 degree‐days is reached—a measure representing the sum of daily mean temperatures above a baseline required for gonadal development (e.g., Colsoul et al., 2021; Maathuis et al., 2020). Based on previous studies and on the molecular detection of *O. edulis* larvae in samples collected on June 15, 2022 samples, we used June 15 as the onset of the spawning period in both 2022 and 2023 simulations, even though the water temperature was slightly below 15°C. Each hour, we released model particles until August 15 to cover the core of the oyster spawning period in the southern North Sea (Maathuis et al., 2020). In total, 292,800 particles were released within a 1‐km radius around each *O. edulis* restoration site per spawning season (Table 1).

To assess regional‐scale connectivity between BRG and SOR, we conducted model experiments releasing particles across the entire BRG, simulating potential larval sources beyond localized restoration sites. In each spawning season, we released 732,000 particles (500 per hour over 61 days) uniformly distributed over BRG. Similar to the sampling season simulations described in the previous section, we conducted both surface and depth‐averaged simulations for the corresponding spawning seasons.

To characterize larval dispersion, we calculated the center of gravity of the newly settled *O. edulis* for each experimental setup, represented by their average latitude and longitude. Additionally, we estimated pairwise connectivity indices between the existing restoration sites and SOR, the entire BRG and SOR, and among individual restoration sites. Connectivity indices were calculated as the percentage of larvae released in a source area arriving at a sink area within the tested pelagic stage duration. For the extended PLD scenario, larvae were considered to have arrived at a sink area if they reached it at least once over the course of the extended PLD period.

## RESULTS

### Molecular detection of *O. edulis*

A total of 262 metabarcoding samples were analyzed, comprising 170 from eDNA and 92 from meroplankton. *O. edulis* was detected in 22 samples: 18 detections in meroplankton and 4 in eDNA samples. These detections occurred across 14 unique stations—at 10 stations based on meroplankton samples and at 4 stations based on eDNA samples (Appendix S1: Table S2). All four eDNA and 2 of the meroplankton detections were from stations sampled during June 15–17, 2022, and 16 meroplankton detections came from stations sampled between June 28 and July 7, 2022.

In the total of 215 qPCR samples (153 from eDNA and 62 from meroplankton), *O. edulis* was detected in 14 samples: 12 detections in meroplankton and 2 in eDNA samples. These detections occurred across 12 unique stations—at 10 stations based on meroplankton samples and at 2 stations based on eDNA samples (Appendix S1: Table S2).

### Temperature regime and wind conditions

**FIGURE 2:**
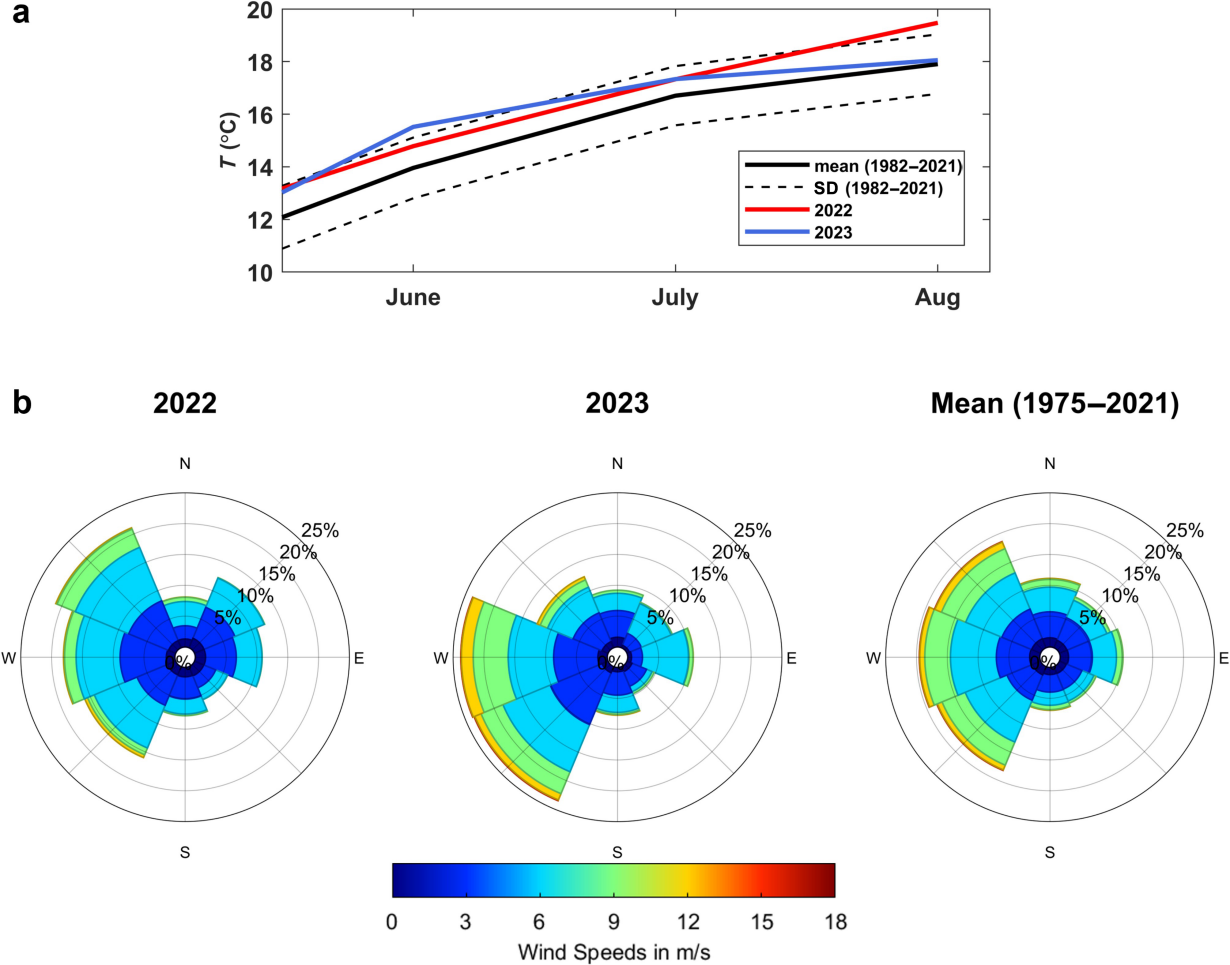
Water temperature and wind conditions in the German Bight in June–August 2022 and 2023 compared to their long‐term mean values. (a) Monthly temperatures in 2022 (red) and 2023 (blue) are shown together with the long‐term mean water temperature (black solid line) and its SD (black dashed lines) calculated for the period from 1982 to 2021. (b) Wind roses show the magnitude and the direction of wind over Borkum Reef Ground in June–August 2022 and 2023 together with the long‐term mean calculated for the period from 1975 to 2021.

Water temperatures in the German Bight during the summers of 2022 and 2023 were higher than the long‐term average estimated over the period 1982–2021 (Figure 2a). However, they generally remained within the SD of the observed long‐term range, except for August 2022 and June 2023, which were 1.5 and 1.6°C above the average, respectively. In 2022, northwesterly and northeasterly winds were more frequent than the 60‐year average, though slightly weaker in strength. In contrast, 2023 showed stronger and more frequent westerly and southwesterly winds (Figure 2b).

### Overlap between modeled and molecularly detected larval distributions

**FIGURE 3:**
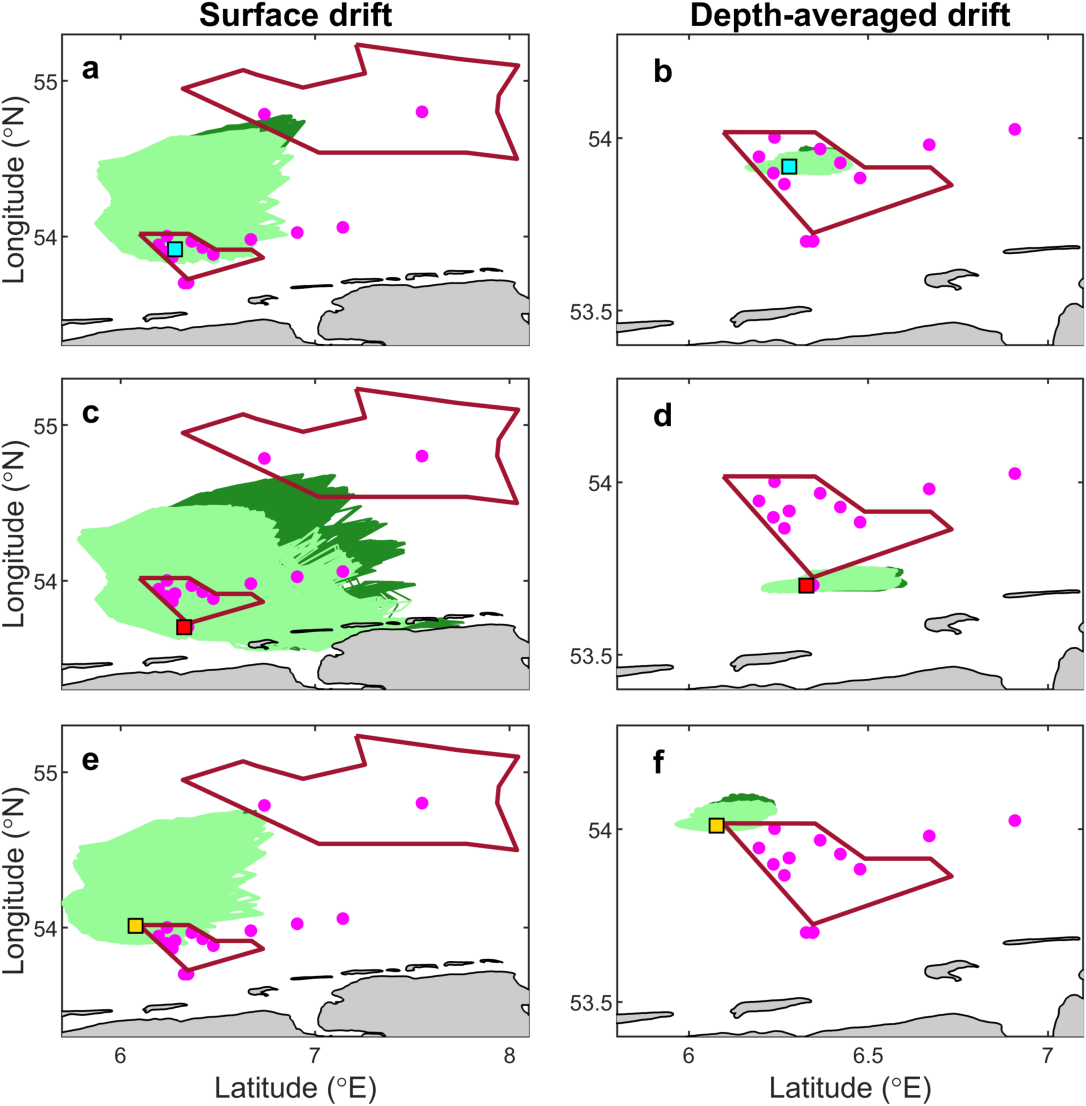
Spatial distribution of modeled larval drift and molecular detections of the European flat oyster during the 2022 sampling campaign (June 15–July 6), shown for both surface (left panels; a, c, and e) and depth‐averaged (right panels; b, d, and f) biophysical model scenarios. Rows of panels represent drift of modeled larvae released over three restoration sites: BRG‐DE (German site in Borkum Reef Ground; top), BRG‐NL (Dutch site south of Borkum Reef Ground; middle), and Gemini‐NL (Dutch site west of Borkum Reef Ground; bottom). The positions of the corresponding restoration sites are shown by squares: cyan for BRG‐DE, red for BRG‐NL, and yellow for Gemini‐NL. Pale green shading represents larval drift under the strict pelagic larval duration (PLD) scenario; dark green shading represents drift under the extended PLD scenario. Magenta squares indicate stations where the European flat oyster was molecularly detected. In panel (e), the extended PLD trajectories (dark green) are not visible, as they did not exceed the spatial extent of the strict PLD trajectory (pale green). Panels (b), (d), and (f) are zoomed‐in views showing only the close vicinity of BRG, because in these specific scenarios, the modeled larvae did not leave the area shown. In panel (d), three magenta symbols are not visible, as they are overlain by the red marker; their positions are in the layer beneath. The red outlines in panels (a), (c), and (e) represent the spatial extent of BRG (southern) and SOR (northern) MPAs.

We found partial but informative correspondence between the spatial distribution of molecular detections and modeled larval dispersal pathways. Notably, a substantial difference was observed for modeled larvae traveling at the surface, as compared to those distributed throughout the water column. The degree of overlap between surface drift scenarios and observed molecular signals varied depending on larval release site and PLD (Figure 3). The strongest overlap was obtained for larvae originating from BRG‐NL (Figure 3c), where 15 out of 17 molecular detections coincided with the predicted larval trajectory. The agreement with observations was less pronounced for larvae originating from BRG‐DE (8 out of 17; Figure 3a) and Gemini‐NL (5 out of 17; Figure 3e).

For the depth‐averaged drift scenario, neither SOR nor the transit region was reached; therefore, we zoomed in on BRG only (Figure 3b,d,f). Few molecular detections within BRG aligned with the predicted larvae dispersal from BRG‐DE (4 out of 17; Figure 3b) and BRG‐NL (3 out of 17; Figure 3d). For Gemini‐NL (Figure 3f), there was no overlap, as all the molecular detections fell outside the modeled larval dispersion.

### Interannual differences in larval dispersal

#### Connectivity between the entire BRG and SOR—Surface drift scenarios

**FIGURE 4:**
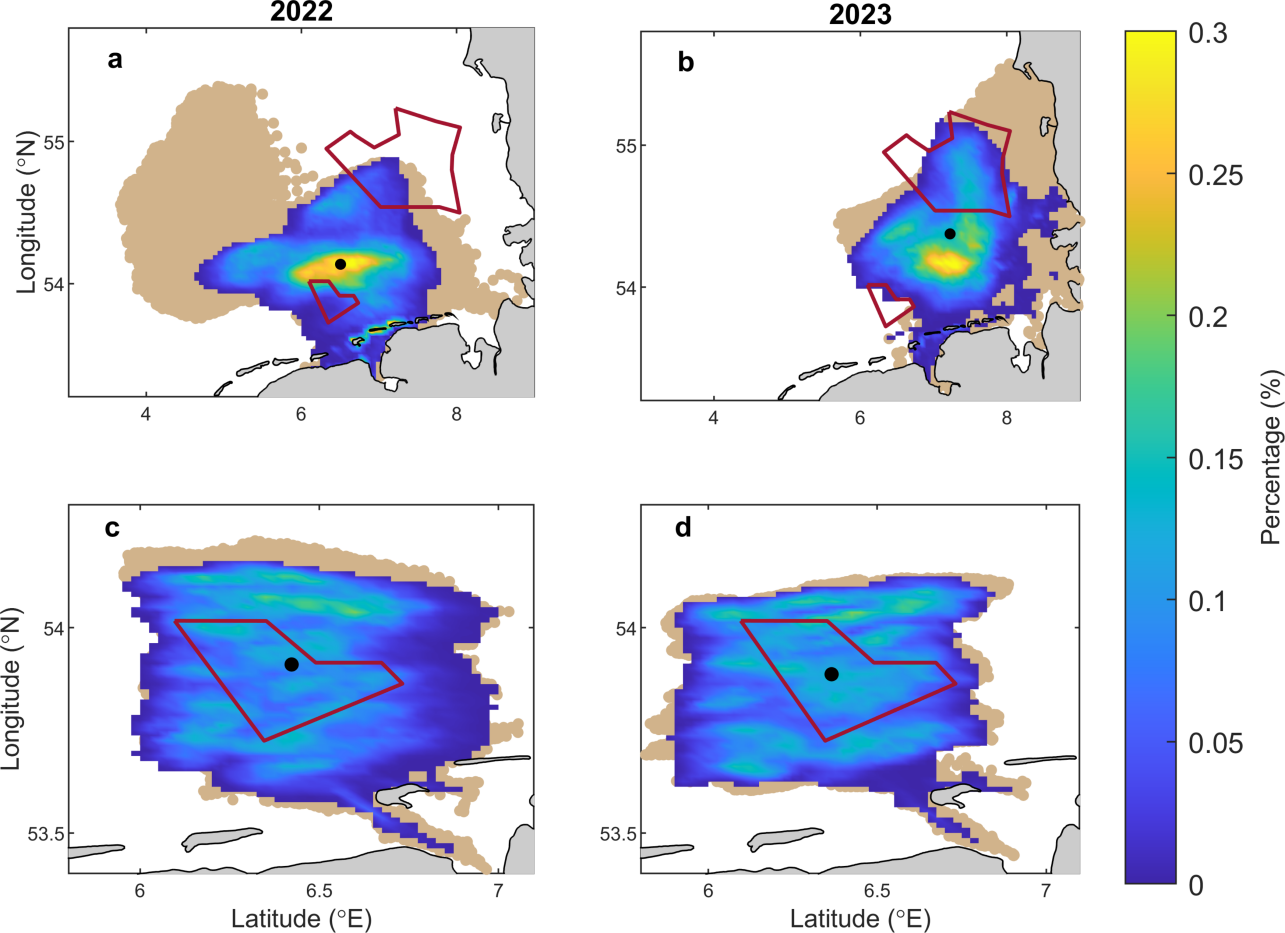
Distribution of the modeled European flat oyster larvae obtained with the surface (a, b) and depth‐averaged (c, d) drift scenario simulations in 2022 (a, c) and 2023 (b, d) for the entire Borkum Reef Ground (BRG). Larval distribution at the end of their strict PLD (blue‐to‐yellow gradient) and their positions within the last two weeks of their extended PLD (brown shaded area) are shown. Blue shades indicate lower densities of larvae in the settlement areas and yellow shades correspond to higher densities of larvae. The black circle is the center of gravity of the modeled larvae at the end of their strict PLD. The red outlines represent the spatial extent of BRG (southern) and Sylt Outer Reef (northern) marine protected areas (MPAs).

In both 2022 and 2023, the strict PLD of the modeled oyster larvae varied between 16 days in June and 11 days in August in the surface scenario. In 2022, larvae released over the entire BRG and assumed to travel in the surface layer dispersed over the western part of the German Bight with some larvae drifting southwards toward the Frisian Islands (Figure 4a). The center of gravity of the modeled larvae at the end of their strict PLD was predicted to be north of BRG (54.136° N, 6.504° E), approximately 24 km away from the center of BRG. The majority of larvae (>97%) were predicted to leave BRG, but only 3% reached SOR (Figure 4a; Table 2). In the extended PLD scenario, there was a more pronounced dispersal of the modeled larvae across the coastal German Bight, compared to the strict PLD scenario (Figure 4a,b). For this scenario, the connectivity index between BRG and SOR was ~10%, almost three times higher than in the strict PLD scenario (Table 2).

**TABLE 2:** Connectivity indices (in % of released particles) obtained in the surface drift scenario in 2022 and 2023.

Year/source		Sink				
		BRG	BRG‐DE	BRG‐NL	Gemini‐NL	SOR
2022						
Source	BRG	2.3 (5.5)	0.2 (0.4)	< 0.1 (0.2)	1.0 (3.5)	3.1 (9.5)
	BRG‐DE	0.7 (0.9)	0.0 (0.0)	0.0 (0.0)	<0.1 (<0.1)	1.0 (9.2)
	BRG‐NL	9.3 (18.4)	<0.1 (0.1)	0.0 (0.0)	0.0 (0.2)	0.0 (2.8)
	Gemini‐NL	1.1 (1.7)	0.0 (0.0)	0.0 (0.0)	0.0 (0.0)	1.0 (6.1)
2023						
Source	BRG	<0.1 (<0.1)	0 (<0.1)	<0.1 (<0.1)	<0.1 (<0.1)	21.7 (74.5)
	BRG‐DE	0.0 (0.0)	0.0 (0.0)	0.0 (0.0)	0.0 (0.0)	27.7 (82.7)
	BRG‐NL	<0.1 (<0.1)	0.0 (0.0)	0.0 (0.0)	< 0.1 (<0.1)	7.8 (61.20)
	Gemini‐NL	0.0 (0.0)	0.0 (0.0)	0.0 (0.0)	0.0 (0.0)	26.2 (78.7)

*Note*: Numbers represent the strict pelagic larval duration (PLD) scenario (with the extended PLD scenario in brackets).

Abbreviations: BRG, entire MPA Borkum Reef Ground; BRG‐DE, German restoration site; BRG‐NL and Gemini‐NL, Netherlands restoration sites; SOR, Sylt Outer Reef.

In 2023, the drift pattern of larvae released in BRG substantially differed from the previous year, with larvae mainly transported northeast and toward the German and Danish coasts of the North Sea (Figure 4b). The center of gravity (54.386° N, 7.246° E) of the newly settled oyster larvae was slightly to the south of SOR, approximately 75.5 km away from BRG. The connectivity indices between BRG and SOR were considerably higher in 2023 than in 2022 and varied between 22% for the strict and 75% for the extended PLD scenarios (Table 2).

#### Connectivity between the entire BRG and SOR—Depth‐averaged scenarios

In the depth‐averaged scenario, the modeled PLD was 1–2 days longer than in the surface scenario, due to the slightly lower depth‐averaged temperature compared to that at the surface. Despite the longer PLD, particles released across the entire BRG in 2022 and 2023 tended to remain localized (Figure 4c,d). A considerable proportion of released particles (26% in the strict PLD scenario and 37% in the extended PLD scenario; Table 3) was predicted to settle within BRG, while the rest were transported outside the MPA, but remained relatively nearby in the transition region. None of the modeled larvae reached SOR (Appendix S1: Figure S1; Table 3).

**TABLE 3:** Connectivity indices (in % of released particles) obtained in the depth‐averaged drift scenario.

Year/source		Sink				
		BRG	BRG‐DE	BRG‐NL	Gemini‐NL	SOR
2022						
Source	BRG	25.7 (36.6)	2.2 (6.1)	2.5 (7.3)	0.9 (1.8)	0.0 (0.0)
	BRG‐DE	95.7 (97.8)	0.9 (2.7)	0.0 (0.0)	0.0 (0.0)	0.0 (0.0)
	BRG‐NL	5.3 (15.8)	0.0 (0.0)	0.2 (0.5)	0.0 (0.0)	0.0 (0.0)
	Gemini‐NL	<0.1 (<0.1)	0.0 (0.0)	0.0 (0.0)	<0.1 (<0.1)	0.0 (0.0)
2023						
Source	BRG	28.7 (39.2)	3.1 (8.0)	2.3 (5.7)	3.3 (7.7)	0.0 (0.0)
	BRG‐DE	79.1 (96.2)	0.0 (0.0)	0.0 (0.0)	0.0 (0.0)	0.0 (0.0)
	BRG‐NL	1.1 (2.7)	0.0 (0.0)	0.3 (1.4)	0.0 (0.0)	0.0 (0.0)
	Gemini‐NL	0.5 (1.4)	0.0 (0.0)	0.0 (0.0)	0.0 (0.0)	0.0 (0.0)

*Note*: Numbers represent the strict pelagic larval duration (PLD) scenario (with the extended PLD scenario in parentheses).

Abbreviations: BRG, entire MPA Borkum Reef Ground; BRG‐DE, German restoration site; BRG‐NL and Gemini‐NL, Netherlands restoration sites; SOR, Sylt Outer Reef.

#### Connectivity between individual restoration sites and SOR

The patterns of larval dispersion obtained for the individual restoration sites are similar to those obtained for the entire BRG. In the 2022 surface drift scenario, BRG‐DE contributed 1% of particles to SOR under the strict PLD and 9% under the extended PLD (Appendix S1: Figure S1; Table 2). Similarly, Gemini‐NL contributed 1% of particles to SOR under the strict PLD and 6% under the extended PLD (Appendix S1: Figure S1; Table 2). BRG‐NL showed no connectivity to SOR under the strict PLD, but increased to 3% under the extended PLD (Table 2). In the 2023 surface drift scenario, there was a dramatic increase in connectivity to SOR compared to 2022. BRG‐DE contributed substantially, with 28% of larvae under the strict PLD, rising substantially to 83% under the extended PLD (Appendix S1: Figure S1; Table 2). Gemini‐NL also saw a notable increase, with 26% of larvae reaching SOR under the strict PLD and 79% under the extended PLD (Appendix S1: Figure S2). BRG‐NL had lower connectivity to SOR, with 8% and 61% of larvae reaching SOR under the strict and extended PLD, respectively (Appendix S1: Figure S3; Table 2).

Depth‐averaged drift simulations for 2022 and 2023 showed no connectivity between individual restoration sites and SOR within either the strict or extended PLD scenarios (Appendix S1: Figures S1–S3; Table 3). Larvae originating from BRG‐DE contributed substantially to the BRG area, with predicted local retention of 96%–98% in 2022 and 79%–96% in 2023. Contributions from BRG‐NL and Gemini‐NL were considerably lower (Table 3). Connectivity among individual restoration sites was consistently low (<1%) in both years and under both PLD scenarios. Self‐recruitment at the scale of individual restoration sites was similarly low, with the exception of the depth‐averaged extended PLD scenario, where BRG‐DE reached 2.7% in 2022 and BRG‐NL 1.4% in 2023 (Table 3).

## DISCUSSION

We investigated the functional connectivity between MPAs in the German North Sea, focusing on potential spillover effects of the European flat oyster (*O. edulis*) from a restoration site in BRG toward SOR. By using DNA metabarcoding of meroplankton, we detected *O. edulis* during the spawning season, confirming the presence of the larvae in the restoration region. Detections from eDNA samples in the water indicate the general presence of *O. edulis*. However, since eDNA consists of genetic traces and does not allow distinction between life stages, these detections could originate from larvae, juveniles, or adults. In contrast, oyster larval presence was inferred directly from meroplankton metabarcoding, since meroplankton were collected using plankton nets and pump‐sieved samples that physically capture planktonic organisms, including *O. edulis* larvae. Biophysical modeling revealed significant interannual variability in larval transport, and even in the surface drift scenario, which resulted in the longest trajectories, the connectivity index between BRG and SOR was only 3% in 2022 due to unfavorable wind conditions. In contrast, more favorable wind conditions in 2023 facilitated a 22% connectivity index between BRG and SOR (Table 2).

### Molecular detection of *O. edulis*

Our findings highlight the complementary suitability of both meroplankton and eDNA samples in identifying *O. edulis*. Particularly, DNA metabarcoding of meroplankton proved effective for monitoring and evaluating connectivity between restored and reference sites. The use of multiple molecular techniques—metabarcoding and qPCR—demonstrated complementary strengths, thereby enhancing the overall detection of *O. edulis*. While metabarcoding offers a broader biodiversity overview and higher sensitivity, it is more prone to false positives due to primer biases, sequencing depth variability, and taxonomic assignment errors (Bylemans et al., 2019; Ficetola et al., 2015; Schenekar et al., 2020). In contrast, qPCR targets taxa‐ or species‐specific DNA sequences and provides more quantitative results, albeit with lower sensitivity (Ficetola et al., 2016; Furlan et al., 2020). Distinguishing between sensitivity and specificity is therefore crucial when interpreting results, and confidence can be improved by employing targeted assays, occupancy modeling (estimating the probability of species presence while accounting for imperfect detection), larger water volumes, and increased biological replication (Allan et al., 2021; Hunter et al., 2019; von Ammon et al., 2023; Zaiko et al., 2022).

The temporal pattern of larval detection, with most positives occurring between late June and early July, aligns with the expected spawning period of *O. edulis* in the study region, which is closely tied to water temperature (Burke et al., 2008; Cano et al., 1997; Chapman et al., 2021; Maathuis et al., 2020). Based on the combination of sample types and molecular methods, our findings suggest that meroplankton samples should be prioritized for detecting *O. edulis* larvae, complemented by eDNA sampling for broader biodiversity assessments. Meroplankton sampling filters larger water volumes, likely increasing detection success, and the combined use of qPCR (specificity) and metabarcoding (sensitivity) provides the most robust and comprehensive assessment.

### Larval dispersal, biophysical modeling, and MPA connectivity

The comparison between observed and modeled larval distributions revealed that dispersal patterns were best explained when larvae were assumed to drift at the surface. Such surface‐drifting behavior has been observed for smaller *O. edulis* larvae (Preston et al., 2020), whereas larger, settlement‐stage larvae have been reported throughout the water column (Wilson, 1987). In contrast, the depth‐averaged scenario accounted only for observations near the restoration sites (Figure 3) and failed to explain occurrences in the transit region or in the SOR. Yet, it remains possible that in the field, a fraction of larvae drift at the surface while others remain deeper in the water column, following different transport pathways (Rodriguez‐Perez et al., 2019).

Additionally, our simulations indicated that larvae observed in BRG likely originated from multiple nearby restoration sites, whereas those detected in the transit region toward SOR were predominantly sourced from the more coastal BRG‐NL site (Figure 3b). However, the modeled drift could not explain the presence of *O. edulis* in SOR during the sampling dates. Backtracking these observations, as conducted by Sidorenko et al. (2025), suggested that larvae may have originated either from SOR itself or from areas in the German Bight south and southwest of SOR, rather than from the more distant BRG. Although Sidorenko et al. (2025) applied a more simplified biophysical model compared to ours, their findings hint at the possible existence of yet‐unidentified *O. edulis* populations or reefs in the German Bight, which may serve as additional larval sources.

Theoretically, new reefs may have begun establishing in recent years through larval spillover from BRG restoration sites, which have been active since 2019. However, such populations have not yet been confirmed through direct observations or monitoring. It is also important to consider the possibility that the larvae detected in SOR originated locally. Depth‐averaged models suggest limited dispersal with stronger larval retention within natal areas, which would support local recolonization of the original or neighboring reefs. However, no known *O. edulis* populations currently exist in SOR, and no resident adults have been observed in this region. In light of this lack of evidence, we favor the hypothesis of distant larval sources. Confirming or excluding a local population in SOR remains a priority for future surveys and could substantially improve our understanding of larval connectivity and population dynamics in this region.

Simulations for the two spawning seasons (2022 and 2023) demonstrated pronounced interannual differences in the dispersal and settlement patterns of *O. edulis* larvae, particularly between the surface and depth‐averaged scenarios. In the surface scenario, larvae dispersed over greater distances, whereas in the depth‐averaged scenario, most larvae released from the three restoration sites in and around BRG were predicted to remain within their natal areas. In this latter scenario, dispersal was primarily driven by tidal currents and only marginally influenced by wind, resulting in relatively consistent patterns between the two years (Figure 4c,d; Appendix S1: Figures S1–S3).

Notably, substantial self‐recruitment rates were predicted across both years within BRG (Table 3), with all three restoration sites contributing larvae to this region. The 1‐km spatial threshold used to define self‐recruitment is already fine‐scale for this purpose, yet may still underestimate settlement at the level of individual restoration sites. The depth‐averaged scenario further highlighted limited dispersal distances, suggesting that a vertical distribution of larvae throughout the water column would primarily promote local retention and the sustainability of nearby populations, while offering little potential for long‐distance dispersal or recolonization of distant oyster beds. This finding complements the surface‐drift scenario, in which longer distance dispersal was possible.

Our results indicate that retention, self‐recruitment, and connectivity between habitats are strongly influenced by the vertical positioning of *O. edulis* larvae. This aligns with previous modeling studies showing that dispersal trajectories are highly sensitive to depth, as different layers of the water column are subject to distinct current velocities and directions (Corell et al., 2012; Gary et al., 2020; Robins et al., 2013; Sundelöf & Jonsson, 2012). However, the vertical distribution of *O. edulis* larvae remains uncertain. Laboratory experiments have reported varying results: Rodriguez‐Perez et al. (2020) observed a preference for near‐bottom positioning, while Preston et al. (2020) found higher concentrations near the surface. These differences may reflect stage‐specific behavior or responses to abiotic factors such as water stratification. Moreover, active vertical migration—a behavior observed in bivalve larvae—could further influence larval positioning and dispersal outcomes (Weinstock et al., 2018). Given the key role of vertical distribution in shaping connectivity, refined experimental or in situ observational approaches are needed to resolve these behavioral influences. Such insights are crucial for optimizing oyster restoration strategies (and similar efforts for other species) and for designing MPA networks that effectively support larval dispersal, retention, recruitment, and long‐term conservation objectives (Smith & Castorani, 2023).

Comparison of observed and modeled larval dispersal suggests that *O. edulis* larvae disperse, at least partly, near the surface. For assessing connectivity between MPAs, the surface scenario is most relevant. A key outcome of this scenario is the pronounced interannual variation in dispersal patterns between 2022 and 2023 (Figure 4a,b). Although water temperature differed slightly between the two spawning seasons, the resulting 1–2 days change in PLD was too minor to explain contrasting dispersal patterns. This indicates that wind‐driven currents were the primary driver of interannual variability. In 2022, weak northerly winds limited northeastward larvae drift (Figure 4a), resulting in low connectivity between BRG and SOR (3%–9%), but higher retention within BRG (2%–5%). In contrast, stronger westerly and southwesterly winds in 2023 enhanced larval transport toward SOR (21%–74%), while retention within BRG dropped below 0.1%. These findings are consistent with Sündermann and Pohlmann (2011), who showed that western and southwestern winds promote cyclonic circulation and eastward water transport in the southern North Sea, whereas winds from other directions can weaken or even reverse this pattern, highlighting the pivotal role of wind in circulation dynamics and, consequently, in larval transport.

In this study, we applied a temperature‐dependent larval drift duration based on estimates of PLD from De Mesel et al. (2018) and Stechele, Hughes, et al. (2023). However, experimental studies indicate that oyster larvae can prolong their pelagic phase by up to two weeks while searching for settlement habitat, effectively doubling their time in the water column. Incorporating this extended phase into our model increased larval dispersal distances and the likelihood of larvae reaching SOR, particularly in 2023 (Table 2). This approach may overestimate connectivity, as it assumes that larvae remain in the water column throughout the full 2‐week period rather than settling earlier. Nonetheless, the results highlight how realistic variability in pelagic duration can substantially influence dispersal and connectivity outcomes (Treml et al., 2015).

Overall, our results show that hydrodynamic and environmental conditions, such as temperature and wind‐driven currents, create the physical framework for dispersal, while larval traits determine the extent and timing of transport and settlement. These findings are consistent with previous studies on other sessile species (Ellien et al., 2004; Robins et al., 2013; Vaz et al., 2013). For European flat oyster restoration, strategies should account for both interannual variability in hydrodynamics and species‐specific biological constraints when aiming to enhance connectivity between MPAs (Rossi et al., 2014).

### Methodological constraints and knowledge gaps

A key limitation of this study is the absence of empirically based knowledge of larval behavior for the biophysical models, which may partly explain the only partial agreement between molecular detections and model‐predicted dispersal patterns of *O. edulis*. Moreover, our sampling covered only the core of the spawning season and thus represents specific larval cohorts rather than all larvae from the full reproductive period. Population success, however, depends on the cumulative dispersal of all larval releases under varying environmental conditions, each contributing differently to connectivity. This highlights the broader challenge of validating biophysical models with empirical field data, particularly for rare or cryptic species (Bode et al., 2019; Swearer et al., 2019).

On the molecular side, detection sensitivity varies between methods. eDNA showed especially low detection rates (~1%–2% of all samples), likely due to limited larval DNA shedding combined with rapid degradation and dilution in seawater (Allan et al., 2021; Wilder et al., 2023). Meroplankton sampling yielded higher detection rates (~19%–20% of all samples). This discrepancy in detection rates may not only be due to the differences in sampling methods, but may partly reflect a true difference in larval abundance, as both methods were not always applied on the same dates and at the same stations.

On the modeling side, simulations based on idealized biological parameters (e.g., temperature‐dependent PLD, no mortality, no behavioral traits) inevitably introduce uncertainties that can either overestimate or underestimate dispersal distances (James et al., 2023). The weaker‐than‐expected concordance between datasets therefore underscores the need to improve both detection strategies and model realism. Future work would benefit from more temporally aligned and spatially intensive sampling, as well as from incorporating larval mortality rates and behavioral traits into biophysical models (Swearer et al., 2019).

A further knowledge gap is the absence of temporally aligned datasets across years. Our 2022 sampling coincided with conditions predicted to limit connectivity between BRG and SOR, favoring larval retention within BRG. Observations from that year were broadly consistent with these predictions, but molecular data from 2023—when conditions favored stronger dispersal—were unavailable. This lack of interannual coverage limits our ability to directly assess year‐to‐year variability in larval transport.

The biological component of the model applied here—specifically the PLD of oyster larvae—was parameterized based on controlled laboratory experiments under ideal, ad libitum feeding conditions (Colsoul et al., 2021; De Mesel et al., 2018; Rodriguez‐Perez et al., 2021; Stechele, Hughes, et al., 2023). In natural environments, however, food availability can be highly variable, and food limitations during the pelagic phase may slow down larval growth and prolong development (Kendall et al., 2013). Such extended pelagic durations could increase dispersal distances and connectivity between source and sink populations (Marshall & Keough, 2003; Strathmann et al., 2008). We partially addressed this uncertainty by comparing strict and extended PLD scenarios, but our model did not include larval mortality, which may be high in oysters (Berntsson et al., 1997; Davis, 1958; Davis & Calabrese, 1969; Robert et al., 1991). Slower growing larvae that remain in the water column for extended periods are more vulnerable to predation, starvation, pollutants, and disease, meaning that even if connectivity is predicted, actual survival may be much lower. Therefore, incorporating realistic growth and mortality rates into biophysical models could enhance their predictive accuracy (Cowen & Sponaugle, 2009; Treml et al., 2012).

Our model treated *O. edulis* larvae as passive particles, with dispersal driven solely by water currents at the surface or averaged over depth. While their swimming speed is negligible compared to the horizontal current velocities, larvae can influence their vertical positioning in the water column (North et al., 2008). Vertical positioning can strongly affect dispersal outcomes, as current velocities and directions vary substantially with depth—a pattern clearly demonstrated by our results for the study region. Neglecting this behavior may therefore limit the accuracy of model predictions, particularly regarding retention, self‐recruitment, and connectivity between different habitats (Cecino & Treml, 2021).

### Implications for oyster restoration and MPA management

Our findings emphasize that restoration efforts should consider not only habitat availability but also variable connectivity shaped by environmental conditions. However, dispersal patterns vary strongly between years, and a reliable annual larval supply cannot be expected from a single site. While 2023 results suggest occasional larval exchange between MPAs, interannual variability underscores the need for management interventions such as translocations or stepping‐stone reefs (Sidorenko et al., 2025; Smith & Castorani, 2023). Therefore, a network of MPAs and restoration locations may be needed to ensure successful reestablishment of *O. edulis*.

The strong interannual contrast in dispersal highlights that restoration strategies must account for environmental fluctuations affecting benthic organisms with meroplanktonic larvae. Some years may favor long‐distance dispersal, enhancing genetic diversity and connectivity between distant populations, while in other years, local recruitment dominates, reinforcing the stability of individual restoration sites (Reynolds et al., 2013). Both processes are essential for the long‐term sustainability of European flat oyster populations in the German Bight. Similar patterns—where self‐recruitment and local retention prevail alongside occasional long‐distance dispersal—have been observed in coral reef fishes (Abecasis et al., 2024; Almany et al., 2007; Hogan et al., 2012; Jones et al., 1999; Pusack et al., 2014). This variability aligns with the concept of dispersal kernels—probability distributions describing larval settlement at various distances from their origin—which can fluctuate in scale, shape, and magnitude across different years and seasons (Catalano et al., 2021). Such fluctuations affect metapopulation dynamics and call for adaptive restoration strategies. Timing interventions to coincide with favorable conditions could improve outcomes: for instance, introducing adult oysters or deploying suitable substrate during years that favor long‐distance larval dispersal may enhance connectivity, whereas in retention‐dominated years, efforts could focus on improving habitat quality and local population density. Coupling operational forecasts with Lagrangian simulations could further support real‐time, adaptive decision making, ultimately strengthening the resilience and sustainability of restored *O. edulis* populations.

In this study, larval release points were based on established restoration sites in the North Sea implemented through national initiatives in The Netherlands and Germany. Although oyster larvae can disperse widely, restoration efforts are typically planned and executed at the national level, influencing site selection, methodologies, and conservation goals. By analyzing larval dispersal from these nationally designated sites, we gain valuable insights into how individual initiatives contribute to broader scale connectivity. These findings underscore the need to internationally align restoration within a cohesive regional strategy, an essential step toward ensuring the long‐term recovery and sustainability of targeted populations, such as the European flat oyster.

## CONCLUSIONS

Our study highlights that functional connectivity between MPAs can be evaluated through the integrated approach presented. In the case of the German Bight, connectivity between BRG and SOR is possible, but irregular and strongly influenced by interannual variability in key environmental factors such as temperature and wind, as well as by the vertical distribution or swimming behavior of meroplanktonic larvae. For the European flat oyster, occasional connectivity between these two MPAs may occur under favorable wind conditions, as observed in 2023, but it still remains uncertain whether natural dispersal alone is sufficient to establish new reefs in SOR. By protecting local recruitment while promoting connectivity, restoration efforts can better support the recovery of *O. edulis* populations and strengthen the ecological integrity of MPAs in the North Sea. Crucially, the integration of biophysical modeling with molecular detections provides a more comprehensive and functional perspective on larval connectivity than either approach alone, delivering transferable insights for oyster restoration and MPA network design.

## AUTHOR CONTRIBUTIONS

Kingsly C. Beng was responsible for methodology, field sampling, molecular analyses, data analysis, and writing the original draft. Anna Akimova contributed to methodology, biophysical modeling, visualization, and writing (review and editing). Silke Laakmann supervised the study and was involved in conceptualization, funding acquisition, molecular analyses, data curation, and writing (review and editing). Vera Sidorenko provided expertise in hydrodynamic modeling and writing (review and editing). Sara Rubinetti focused on wind dynamics modeling, data curation, and writing (review and editing). Santiago E. A. Pineda‐Metz assisted with methodology, field sampling, and writing (review and editing). Bernadette Pogoda contributed to conceptualization, funding acquisition, and writing (review and editing). Sarah C. Brand was involved in methodology, molecular analyses, data curation, and writing (review and editing). Kerstin Klemm, K. Mathias Wegner, and Lisa N. S. Shama each contributed to methodology, molecular analyses, and writing (review and editing). Lara Schmittmann, Luis Gimenez, Katharina Alter, and Brecht Stechele participated in writing (review and editing). Amin Rahdarian was responsible for visualization and writing (review and editing). Christian Winter and Inna Sokolova contributed to writing (review and editing). Alexey Androsov provided expertise in hydrodynamic modeling and writing (review and editing). Anne F. Sell supervised the study and contributed to conceptualization, funding acquisition, and writing (review and editing).

## CONFLICT OF INTEREST STATEMENT

The authors declare no conflicts of interest.

## Supporting information


Appendix S1.

## Data Availability

Raw sequence data and sample metadata are available in the European Nucleotide Archive (ENA) under accession no. PRJEB98425 at https://www.ebi.ac.uk/ena/browser/view/PRJEB98425.
