# Supplementary material for: Integrating molecular methods and biophysical modeling to assess functional connectivity between marine protected areas
Source: Ecol Appl. 2025 Dec 8;35(8):e70150. doi: 10.1002/eap.70150 (PMC12683707; doi:10.1002/eap.70150)
Supplement: Supplementary file 1 — Appendix S1. [file EAP-35-e70150-s001.pdf]

Appendix S1

Integrating molecular methods and biophysical modeling to assess functional connectivity between marine protected areas

Kingsly C. Beng, Anna Akimova, Silke Laakmann, Vera Sidorenko, Sara Rubinetti, Santiago E. A. Pineda-Metz, Bernadette Pogoda, Sarah C. Brand, Kerstin Klemm, K. Mathias Wegner, Lisa N. S. Shama, Lara Schmittmann, Luis Gimenez, Katharina Alter, Brecht Stechele, Amin Rahdarian, Christian Winter, Alexey Androsov, Inna Sokolova, Anne F. Sell

Ecological Applications

Supplementary tables

Table S1. Primers and TaqMan probes targeting O. edulis 16S rRNA and general Bivalvia 18S rRNA genes

	O. edulis 16S rRNA	General Bivalvia 18S rRNA
Forward primer	5´- GGCGCCCCACCTAAAAAT -3´	5´- AGCCACACGAGATTGAGCAAT -3´
Reverse primer	5´- AGACCCCGTGCAACTTTTAAAG -3´	5´- GCGGCCCCGAACATCTA -3´
Probe	5´- FAM- TGAAACTCCTAAACAAGTTG-MGB	5´- CY5-ACAGGTCTGTGATGCC-MGB

Table S2. Summary of *Ostrea edulis* detections by method, sample type, and station coverage

Method	Sample type	Samples analyzed	Positive detections	Detection rate (%)	Stations with detections
Metabarcoding	Meroplankton	92	18	19.6	10
	eDNA	170	4	2.4	4
qPCR	Meroplankton	62	12	19.4	10
	eDNA	153	2	1.3	2

Supplementary figures

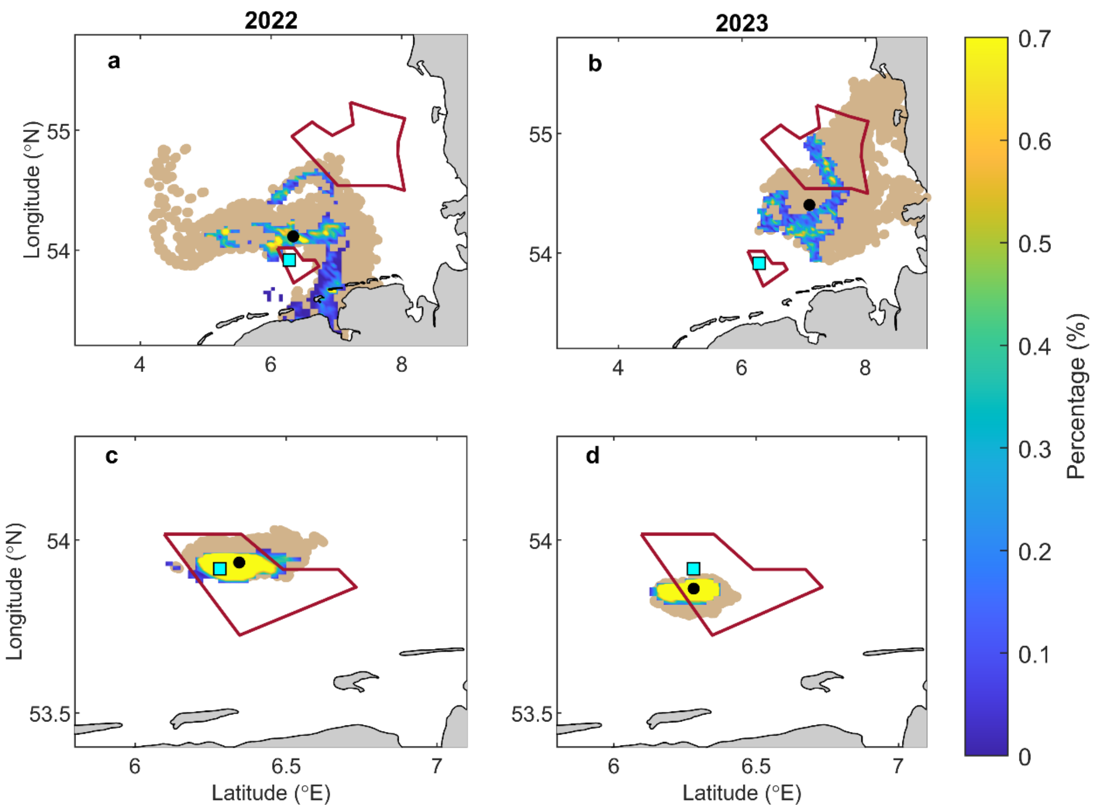

Figure S1. Distribution of the modeled European flat oyster larvae obtained with the surface (a, b) depth-averaged (c, d) drift scenario simulations in 2022 (a, c) and 2023 (b, d), with larvae released within a circle of 1 km over the current BRG-DE restoration site (cyan square). Larval distribution at the end of their strict pelagic larval duration (PLD) (blue-to-yellow gradient) and positions within the last two weeks of their extended PLD (brown shaded area) are shown. Blue shades indicate lower densities of larvae in the settlement areas and yellow shades correspond to higher densities of larvae. The black circle is the center of gravity of the modeled larvae at the end of their strict PLD. The red outlines represent the spatial extent of BRG (southern) and SOR (northern) MPAs.

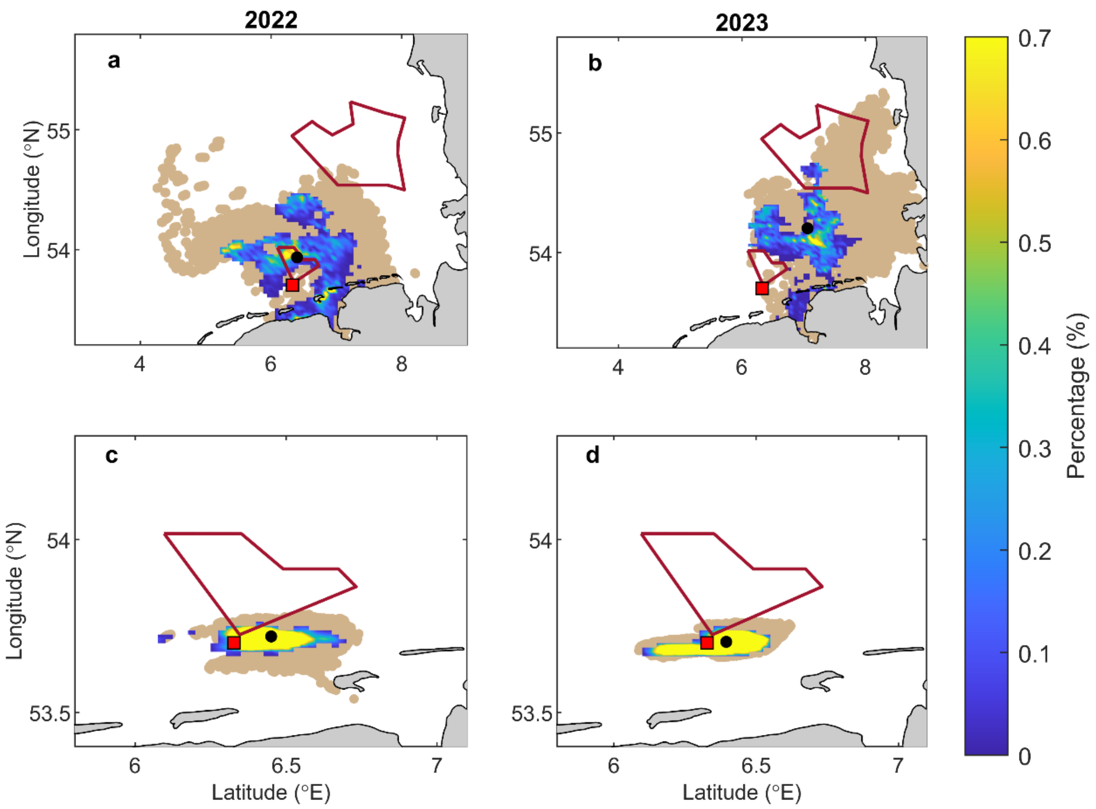

Figure S2. Distribution of the modeled European flat oyster larvae obtained with the surface (a, b) depth-averaged (c, d) drift scenario simulations in 2022 (a, c) and 2023 (b, d), with larvae released within a circle of 1 km over the current BRG-NL restoration site (red square). Larval distribution at the end of their strict pelagic larval duration (PLD) (blue-to-yellow gradient) and positions within the last two weeks of their extended PLD (brown shaded area) are shown. Blue shades indicate lower densities of larvae in the settlement areas and yellow shades correspond to higher densities of larvae. The black circle is the center of gravity of the modeled larvae at the end of their strict PLD. The red outlines represent the spatial extent of BRG (southern) and SOR (northern) MPAs.

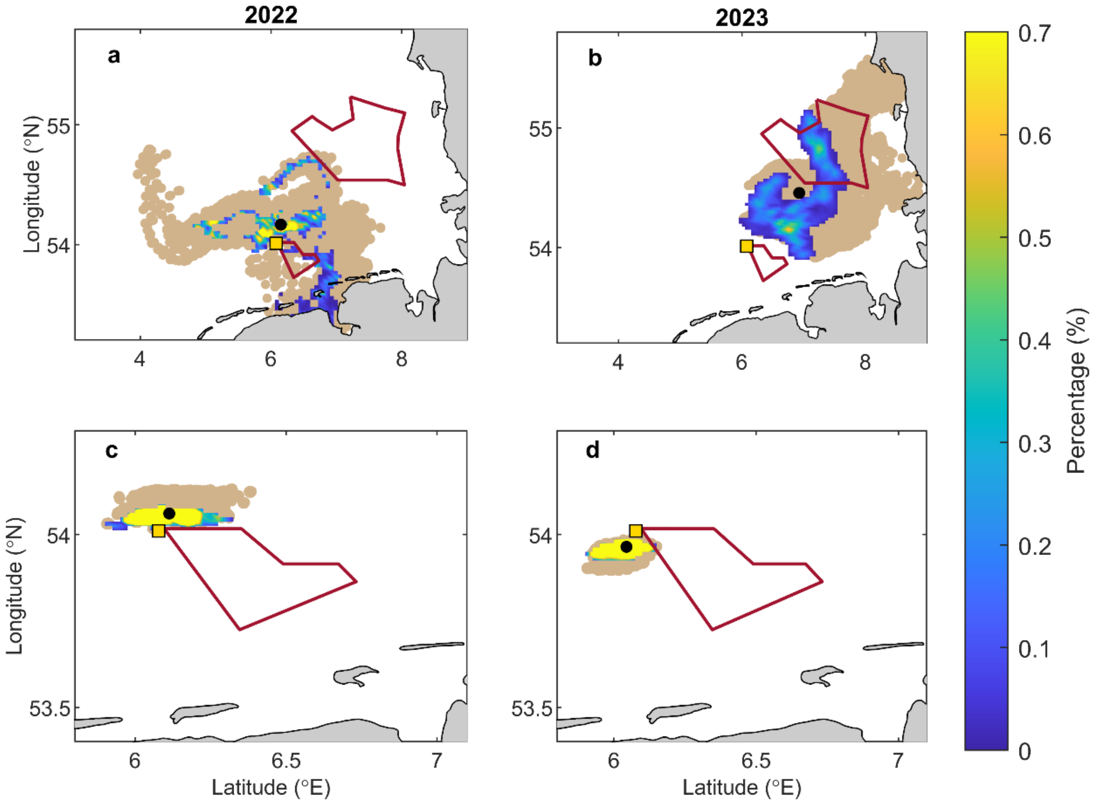

Figure S3. Distribution of the modeled European flat oyster larvae obtained with the surface (a, b) depth-averaged (c, d) drift scenario simulations in 2022 (a, c) and 2023 (b, d), with larvae released within a circle of 1 km over the current Gemini-NL restoration site (yellow). Larval distribution at the end of their strict pelagic larval duration (PLD) (blue-to-yellow gradient) and positions within the last two weeks of their extended PLD (brown shaded area) are shown. Blue shades indicate lower densities of larvae in the settlement areas and yellow shades correspond to higher densities of larvae. The black circle is the center of gravity of the modeled larvae at the end of their strict PLD. The red outlines represent the spatial extent of BRG (southern) and SOR (northern) MPAs.
